# A comprehensive, population level evaluation of previously reported drug triggers of pemphigus highlights immunomodulatory capacity as a common characteristic

**DOI:** 10.3389/fimmu.2024.1508129

**Published:** 2025-01-21

**Authors:** Justin Baroukhian, Kristina Seiffert-Sinha, Animesh A. Sinha

**Affiliations:** Department of Dermatology, Jacobs School of Medicine and Biomedical Sciences, Buffalo, NY, United States

**Keywords:** drug, medication, pemphigus, autoimmunity, exposome, FAERS, environmental factors, autoimmune bullous disease

## Abstract

**Question:**

Can previously reported, largely anecdotal associations between exposure to any of a comprehensive list of putative trigger drugs and the development of pemphigus be reproduced using population level data?

**Findings:**

In this series of observational, retrospective, case-control, pharmacovigilance analyses of the FDA Adverse Event Reporting System, the odds of reporting the adverse event pemphigus were significantly elevated among individuals exposed to 11/36 previously reported trigger drugs namely, gold sodium thiomalate, penicillamine, piroxicam, rifampin, hydroxychloroquine, imiquimod, hydrochlorothiazide, irbesartan, lisinopril, nivolumab, and nifedipine.

**Meaning:**

Environmental exposures such as drugs are relevant players in the pathogenesis of autoimmune diseases and clinicians who treat patients with autoimmune blistering diseases such as pemphigus should consider performing a detailed medication history leveraging this information regarding deleterious drug-disease interactions at initial evaluation as well as longitudinal monitoring of patients to better inform clinical care decisions.

**Importance:**

Pemphigus vulgaris (PV) is a rare, potentially fatal autoimmune disease with pathogenic contributions from both genetic as well as environmental factors, notably drug exposures. Despite anecdotal reports linking multiple drugs to PV, corroborating evidence from large datasets is missing.

**Objective:**

To examine the extent to which previously reported associations between a comprehensive list of 36 drugs implicated in PV pathogenesis could be replicated using population-level pharmacovigilance data.

**Design:**

Series of observational, retrospective, case-control, pharmacovigilance analyses (one analysis/drug, 36 total).

**Setting:**

Population based.

**Participants:**

Individuals who submitted a report of a drug-related adverse event to the FDA from Q4 of 2003 to Q2 of 2023.

**Exposure:**

Cases were identified by the presence of adverse events described by the MedDRA preferred term “pemphigus” (10034280) and then sorted based on exposure to each of the drugs of interest.

**Main outcomes and measures:**

Reporting Odds Ratios (RORs) quantifying the association between a given drug exposure and reports of pemphigus adverse events.

**Results:**

The analyses revealed statistically significant associations between reports of pemphigus and exposure to 11/36 previously reported drugs, two of which had particularly high RORs (>200) [gold sodium thiomalate (ROR, 266.0; 95% CI, 202.6-349.3) and hydroxychloroquine (ROR, 282.6; 95% CI, 261.0-306.1)], three had very strong RORs (14-45) [penicillamine (ROR, 30.5; 95% CI, 11.4-81.7), piroxicam (ROR, 14.8; 95% CI, 8.2-26.7), and imiquimod (ROR, 42.3; 95% CI, 26.2-68.3)], and six had modestly strong RORs (2-5) [rifampin (ROR, 2.8; 95% CI, 1.4-5.6), hydrochlorothiazide (ROR, 1.6; 95% CI, 1.2-2.1), irbesartan (ROR, 2.7; 95% CI, 1.6-4.4), lisinopril (ROR, 5.3; 95% CI, 4.5-6.2), nivolumab (ROR, 2.7; 95% CI, 1.8-4.1), and nifedipine (ROR, 3.0; 95% CI, 1.9-5.0)]. Associations for other previously reported drugs (25/36) were not detected.

**Conclusions and relevance:**

This study represents a comprehensive evaluation of suspected drug triggers of pemphigus using real-world data. The significant associations reported here provide empirical support for the hypothesis that certain drugs act as triggers for PV. Moreover, all of the drugs found to be associated with PV in this study harbor immunomodulatory capacity, suggesting that the ability to induce such perturbations, directly or indirectly, may be a critical factor connecting drug exposure to pemphigus pathogenesis. However, the absence of signals for other previously reported putative trigger drugs does not preclude their potential role in PV pathogenesis. Our findings reinforce the need for larger, more definitive studies to confirm these associations and to explore the mechanisms by which these drugs may contribute to PV development. Finally, these findings underscore the importance of considering environmental factors in the development and course of PV in genetically susceptible individuals.

## 1 Introduction

The genesis of autoimmune disorders is recognized to be the result of a complex composite of both genetic and environmental influences, including potential pharmacologic triggers, acting in concert to disrupt immune self-tolerance (1). Pemphigus vulgaris (PV), a rare, potentially fatal autoimmune disorder characterized by the production of autoantibodies against desmosomal proteins, particularly desmoglein 3 (Dsg3) and desmoglein 1 (Dsg1), serves as a classic example of an organ-specific autoimmune disease where significant genetic predisposition is well established. These autoantibodies impair cell-cell adhesion within the epidermis, causing acantholysis and giving rise to the formation of flaccid blisters and erosions on the skin and mucous membranes (2). While Dsg3 and Dsg1 are central to PV pathogenesis, recent studies have identified additional autoantibody targets (3–7), underscoring the multifactorial mechanisms driving disease progression.

The multifaceted interplay of factors influencing susceptibility to PV is reflected in its epidemiological patterns, which demonstrate significant variations in incidence, prevalence, and average age of onset across different geographical regions. These disparities are thought to result from a combination of genetic variability and diverse environmental exposures, and likely further shaped in part by the unique sociocultural and material contexts of each locale. Reported prevalence of PV ranges from a low of 0.38/100,000 individuals in Bulgaria to 30/100,000 in Iran (8). As with many autoimmune diseases, PV is often associated with additional co-morbid autoimmune conditions both in patients themselves as well as among their first-degree family members (9). An overwhelming majority of Caucasian PV patients carry either the DRB1*0402 or DQB1*0503 class II HLA alleles (10–14). However, genetic predisposition alone cannot account for disease onset in any given individual, as evidenced by the incomplete concordance observed in monozygotic twin studies in autoimmune diseases including multiple sclerosis and rheumatoid arthritis (15–17). This brings to the forefront the “exposome”, a term encapsulating the multitude of environmental and lifestyle factors—including medications, infections, psychosocial stressors, dietary factors, immunizations, and physical insults —that collectively contribute to PV etiopathogenesis (1, 18, 19). A thorough review of the literature reveals that drugs and pharmaceuticals are repeatedly identified as critical environmental triggers for PV (20, 21), with a recent analysis by our group determining that they represent the plurality (35%) of all research on environmental triggers in PV (22).

**Figure 1:**
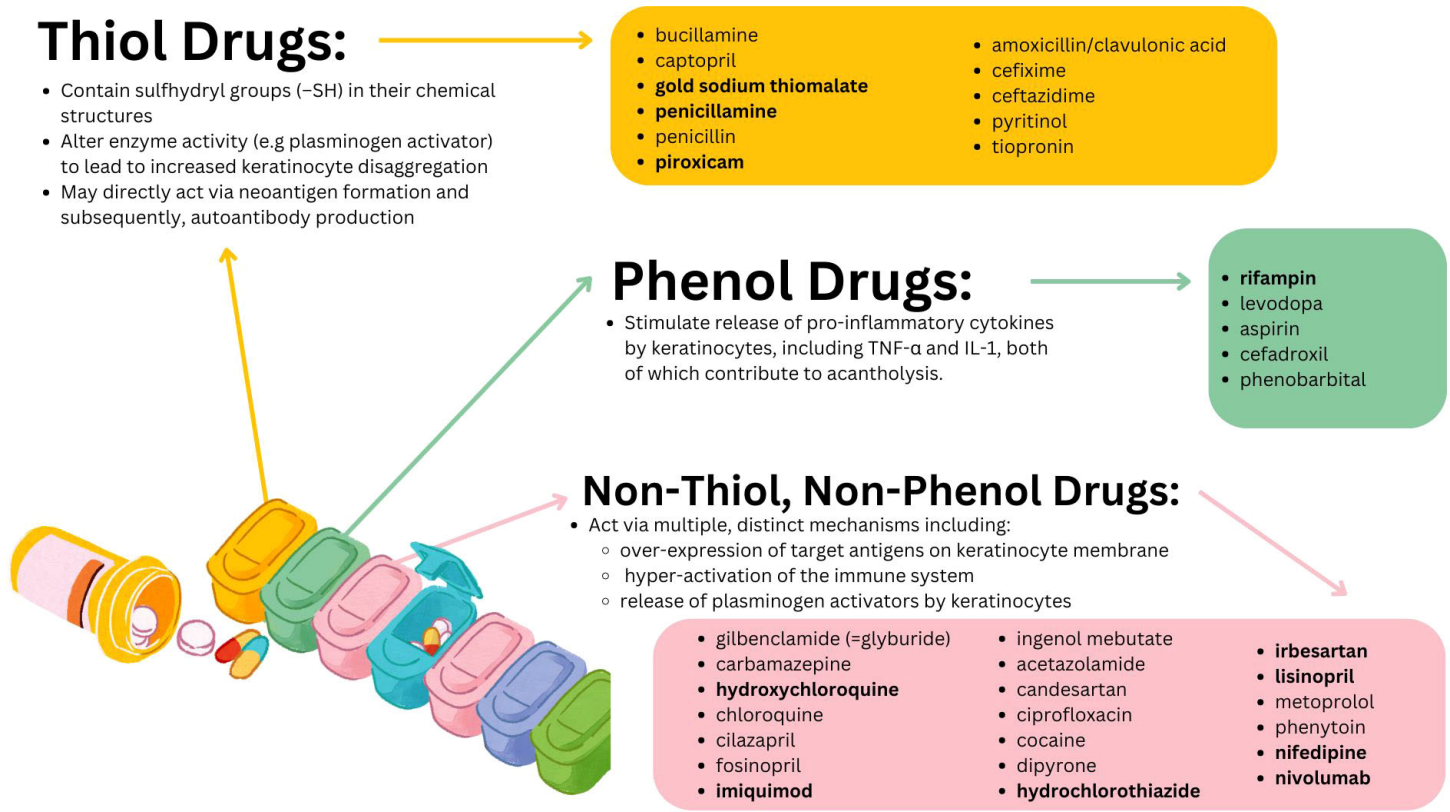
Purported mechanisms of action as well as the individual constituents of the three major classes of putative PV triggering drugs. Drug names in bold are those for whom a significant pharmacovigilance signal was detected in the present work.

The first report of pemphigus triggered by pharmaceutical use in the literature was made by Degos et al. who in 1969 described a case of pemphigus triggered by penicillamine in a patient being treated for Wilson disease (23). Later, a 1976 study found that about 7% of patients taking penicillamine for at least 6 months went on to develop pemphigus (24). Subsequent work has brought to light a diverse range of agents implicated in the initiation and intensification of pemphigus. These compounds are typically categorized by virtue of common biochemical or structural properties, specifically into the thiol, phenol, and non-thiol non-phenol groups (1, 20, 21). Each group is associated with unique purported mechanisms in provoking pemphigus [illustrated in **Figure 1** and expounded upon further elsewhere (1, 20, 21)]. Among those drugs associated with triggering PV are agents as common as lisinopril [non-thiol, non-phenol group (22)] or aspirin [phenol group (22)] and as obscure as gold sodium thiomalate [thiol group (22)]. Presently, the links between most medications and pemphigus are derived from isolated case reports (23–28) which, while valuable, are inherently limited in terms of the evidentiary weight they can offer by virtue of the anecdotal and idiosyncratic nature of case reports.

The World Health Organization (WHO) characterizes pharmacovigilance as the scientific field focused on the identification, evaluation, comprehension, and mitigation of adverse drug reactions and related issues (29). The FDA Adverse Event Reporting System (FAERS), maintained by the Food and Drug Administration (FDA), is a pharmacovigilance resource which collects reports of adverse events associated with drug and therapeutic biologic products and is designed to support drug safety monitoring in the aftermarket period (29). FAERS includes both voluntary submissions from healthcare professionals and consumers, and mandatory reports from manufacturers. As the largest such spontaneous reporting database globally, with over 11 million entries at authorship (30), disproportionality analysis of reporting in FAERS is crucial for untangling whether observed patterns of drug-adverse event reports are merely coincidental or, in fact, drug-induced. This approach is particularly valuable for detecting rare AEs that may not appear during pre-approval clinical trials.

Given the growing evidence in the literature supporting the role of environmental factors in shaping the onset and course of disease in pemphigus, the outsized place of drugs among those implicated environmental factors, and the paucity of population level data on either, we conducted a series of pharmacovigilance analyses with the intention of uncovering additional evidence to support the link between particular pharmaceutical agents previously reported to trigger PV and the development of pemphigus using publicly available, FDA-generated data. We found statistically significant, disproportionately elevated reporting of the drug-adverse event combinations of 11 out of 36 drugs investigated and pemphigus which will be detailed below, each of which constitute pharmacovigilance “signals” worthy of further investigation.

## 2 Methods

### 2.1 Study design and data sources

This observational, retrospective, pharmacovigilance analysis used the FAERS database to analyze the relationship between the adverse event of “pemphigus” and exposure to any of the drugs previously reported across multiple systematic reviews (20, 22) to be associated with triggering PV (listed in **Table 1**). We employed the validated pharmacovigilance tool OpenVigil 2.1 (accessed on 11/13/2023) to query the FAERS database and perform disproportionality analysis. This study involves FAERS data from Q4 of 2003 to Q2 of 2023.

**Table 1:** Complete listing of ATC^a^ 1 and 2 categorizations for each drug analyzed.

Drug^b^	ATC 1	ATC 1 - Text	Number of Drugs in ATC-1 Category (%)^c^	ATC 2	ATC 2 - Text	Number of Drugs in ATC-2 Category (%)^c^
Gilbenclamide (=glyburide)	A	ALIMENTARY TRACT AND METABOLISM	1/36 (2.78)	A10	DRUGS USED IN DIABETES	1/36 (2.78)
Acetylsalicylic acid^d^ (aspirin)	B	BLOOD AND BLOOD FORMING ORGANS	1/36 (2.78)	B01	ANTITHROMBOTIC AGENTS	1/36 (2.78)
**Hydrochlorothiazide**	C	CARDIOVASCULAR SYSTEM	9/36 (25)	C03	DIURETICS	1/36 (2.78)
Metoprolol	C	CARDIOVASCULAR SYSTEM		C07	BETA BLOCKING AGENTS	1/36 (2.78)
**Nifedipine**	C	CARDIOVASCULAR SYSTEM		C08	CALCIUM CHANNEL BLOCKERS	1/36 (2.78)
**Lisinopril**	C	CARDIOVASCULAR SYSTEM		C09	AGENTS ACTING ON THE RENIN-ANGIOTENSIN SYSTEM	6/36 (16.67)
**Irbesartan**	C	CARDIOVASCULAR SYSTEM		C09	AGENTS ACTING ON THE RENIN-ANGIOTENSIN SYSTEM	
Captopril	C	CARDIOVASCULAR SYSTEM		C09	AGENTS ACTING ON THE RENIN-ANGIOTENSIN SYSTEM	
Cilazapril	C	CARDIOVASCULAR SYSTEM		C09	AGENTS ACTING ON THE RENIN-ANGIOTENSIN SYSTEM	
Fosinopril	C	CARDIOVASCULAR SYSTEM		C09	AGENTS ACTING ON THE RENIN-ANGIOTENSIN SYSTEM	
Candesartan	C	CARDIOVASCULAR SYSTEM		C09	AGENTS ACTING ON THE RENIN-ANGIOTENSIN SYSTEM	
**Imiquimod**	D	DERMATOLOGICALS	2/36 (5.56)	D06	ANTIBIOTICS AND CHEMOTHERAPEUTICS FOR DERMATOLOGICAL USE	2/36 (5.56)
Ingenol Mebutate	D	DERMATOLOGICALS		D06	ANTIBIOTICS AND CHEMOTHERAPEUTICS FOR DERMATOLOGICAL USE	
Tiopronin	G	GENITO URINARY SYSTEM AND SEX HORMONES	1/36 (2.78)	G04	UROLOGICALS	1/36 (2.78)
Penicillins	J	ANTIINFECTIVES FOR SYSTEMIC USE	7/36 (19.44)	J01	ANTIBACTERIALS FOR SYSTEMIC USE	6/36 (16.67)
Amoxicillin/Clavulonate	J	ANTIINFECTIVES FOR SYSTEMIC USE		J01	ANTIBACTERIALS FOR SYSTEMIC USE	
Cefixime	J	ANTIINFECTIVES FOR SYSTEMIC USE		J01	ANTIBACTERIALS FOR SYSTEMIC USE	
Ceftazidime	J	ANTIINFECTIVES FOR SYSTEMIC USE		J01	ANTIBACTERIALS FOR SYSTEMIC USE	
Cefadroxil	J	ANTIINFECTIVES FOR SYSTEMIC USE		J01	ANTIBACTERIALS FOR SYSTEMIC USE	
Ciprofloxacin	J	ANTIINFECTIVES FOR SYSTEMIC USE		J01	ANTIBACTERIALS FOR SYSTEMIC USE	
**Rifampin**	J	ANTIINFECTIVES FOR SYSTEMIC USE		J04	ANTIMYCOBACTERIALS	1/36 (2.78)
**Nivolumab**	L	ANTINEOPLASTIC AND IMMUNOMODULATING AGENTS	1/36 (2.78)	L01	ANTINEOPLASTIC AGENTS	1/36 (2.78)
**Gold sodium thiomalate**	M	MUSCULO-SKELETAL SYSTEM	4/36 (11.11)	M01	ANTIINFLAMMATORY AND ANTIRHEUMATIC PRODUCTS	4/36 (11.11)
**Penicillamine**	M	MUSCULO-SKELETAL SYSTEM		M01	ANTIINFLAMMATORY AND ANTIRHEUMATIC PRODUCTS	
**Piroxicam**	M	MUSCULO-SKELETAL SYSTEM		M01	ANTIINFLAMMATORY AND ANTIRHEUMATIC PRODUCTS	
Bucillamine	M	MUSCULO-SKELETAL SYSTEM		M01	ANTIINFLAMMATORY AND ANTIRHEUMATIC PRODUCTS	
Cocaine	N	NERVOUS SYSTEM	7/36 (19.44)	N01	ANESTHETICS	1/36 (2.78)
Dipyrone	N	NERVOUS SYSTEM		N02	ANALGESICS	1/36 (2.78)
Phenobarbital	N	NERVOUS SYSTEM		N03	ANTIEPILEPTICS	3/36 (8.33)
Carbamazepine	N	NERVOUS SYSTEM		N03	ANTIEPILEPTICS	
Phenytoin	N	NERVOUS SYSTEM		N03	ANTIEPILEPTICS	
Levodopa	N	NERVOUS SYSTEM		N04	ANTI-PARKINSON DRUGS	1/36 (2.78)
Pyritinol	N	NERVOUS SYSTEM		N06	PSYCHOANALEPTICS	1/36 (2.78)
**Hydroxychloroquine**	P	ANTIPARASITIC PRODUCTS, INSECTICIDES AND REPELLENTS	2/36 (5.56)	P01	ANTIPROTOZOALS	2/36 (5.56)
Chloroquine	P	ANTIPARASITIC PRODUCTS, INSECTICIDES AND REPELLENTS		P01	ANTIPROTOZOALS	
Acetazolamide	S	SENSORY ORGANS	1/36 (2.78)	S01	OPHTHALMOLOGICALS	1/36 (2.78)

a ATC, The Anatomical Therapeutic Chemical (ATC) classification system of the World Health Organization (WHO). The drugs evaluated in this work were classified according to the WHO’s Anatomical Therapeutic Chemical (ATC) classification system. Classification in the ATC system consists of 5 levels wherein drugs are grouped, at various levels, according to the organ or system on which they act and their therapeutic, pharmacological and chemical properties. The first level of ATC classification (ATC 1) is concerned with the main anatomical or pharmacological group a drug acts on, for example C, cardiovascular system or M, musculo-skeletal system (31). The second level of ATC classification (ATC 2) is concerned with a drug’s pharmacological or therapeutic subgroup, for example C08 = calcium channel blockers or M01 = anti-inflammatory and anti-rheumatic products (31). The 3rd, 4th, and 5th levels of ATC classification include levels of specificity beyond that which is appropriate or meaningful in the present work.

b Cells in this column whose backgrounds are green and text boldfaced are those of drugs for which a positive pharmacovigilance signal with pemphigus was detected in the present analysis, see Results.

c The color of the cells in these columns correspond to the proportion of all drugs evaluated herein represented by a particular ATC 1 or ATC 2 group, with:

<5% - yellow, 5-9.99% - orange, 10-14.99% - red, 15-19.99% - light purple, and ≥20% - dark purple.

d Multiple ATC classifications exist for acetylsalicylic acid.

Adverse events in FAERS, as queried in the present study, are reported in accordance with the Medical Dictionary for Regulatory Activities (MedDRA) version 24, as previously described (32).

### 2.2 Case selection and controls

Cases were identified by the presence of the MedDRA Preferred Term (PT) for “pemphigus” (10034280, which includes all members of the pemphigus group of diseases) and then sorted based on exposure to a drug of interest, or lack thereof, and this process was then repeated for each of the drugs previously reported to trigger PV. The comparator or control used to determine whether a drug-event combination of interest is disproportionately overrepresented is all other reports of adverse events in the database, excluding the adverse event of interest (33–35).

### 2.3 Source(s) of putative PV trigger drugs evaluated

The list of putative PV trigger drugs examined in this work were drawn from two recent systematic reviews which assessed the identity and role of all known environmental factors, including drugs, reported to influence the onset and/or course of PV [2022 by Adebiyi et al. (22) and 2018 by Tavakolpour (20)]. Inclusion of any given drug in *either* of the two works was sufficient to constitute inclusion in the present analysis. However, there were certain agents reported in those reviews which, upon further examination, proved not to be pharmacological agents, drugs, or medicines but rather chemical or physical agents to which humans may be exposed. Specifically, those agents - not included in the present work, which is concerned exclusively with *drug* triggers of PV - were: pentachlorophenol (an organochlorine used as a pesticide and wood preservative), diazinon (an organophosphate insecticide), and methylisothiazolinone (a biocidal preservative agent used in personal care products).

Apart from reviews like those cited above, based on the largely anecdotal body of literature regarding the role of environmental factors in disease onset and intensification in PV, to our knowledge, there are no examples of methodologically rigorous investigations (e.g. those with comparison groups or large sample sizes) of the caliber required to modify clinical practice in existence to date.

### 2.4 Statistical analysis

Calculations of the RORs and their 95% CIs were performed within OpenVigil 2.1, as previously described (36, 37). The ROR assesses the disproportionality of adverse event reporting for a specific drug, comparing the odds of the event occurring with the drug of interest to the odds with all other drugs in the database. A ROR of 1 indicates no association, while values greater than 1 suggest a potential signal, with higher values indicating a stronger association. A potential drug-adverse event combination is deemed significant (a “positive signal”) when the lower bound of the ROR’s 95% CI exceeds 1, and the chi-square (χ²) value is greater than 4, and the number of reports (n) exceeds 3 (33, 34, 38, 39). These thresholds are meant to ensure the reliability of the findings by reducing the likelihood of spurious associations due to random variation. This approach is widely used in pharmacovigilance to identify potential drug-event associations, though additional methods are also employed (39).

## 3 Results

### 3.1 Over sixty percent of putative PV trigger drugs are related to either the cardiovascular system, the nervous system, or systemic anti-infective agents

Qualitative review of the complete set of drugs previously reported in the literature to trigger PV and evaluated in this work (n=36) reveals that the majority (23, 63.88%) of agents are, based on their first-level ATC classifications, those associated with: the cardiovascular system (9, 25.00%), the nervous system (7, 19.44%), or anti-infectives for systemic use (7, 19.44%). First level ATC classifications for all drugs evaluated in this work are listed in **Table 1**.

### 3.2 One out of four drugs reported to trigger PV are anti-hypertensive agents of diverse pharmacologic classes

Considering the significant clinical associations of the drugs reviewed herein, anti-hypertensive agents of various pharmacological classes, as an aggregate, constitute the most common subgroup of drugs implicated in triggering PV. These various classes of anti-hypertensive drugs (9, 25.00%) include: agents acting on the renin-angiotensin system (6, 16.67%), calcium channel blockers (1, 2.78%), diuretics (1, 2.78%), and beta-blocking agents (1, 2.78%). However, in terms of individual second-level ATC classifications, antibacterials for systemic use (6, 16.67%) are tied with agents acting on the renin-angiotensin system (6, 16.67%) as the largest *single* subgroup of implicated drugs. Second level ATC classifications for all drugs evaluated in this work are listed in **Table 1**.

### 3.3 Pharmacovigilance analysis provides additional evidence for a subset of both previously well established as well as lesser reported PV trigger drugs

**Figure 2:**
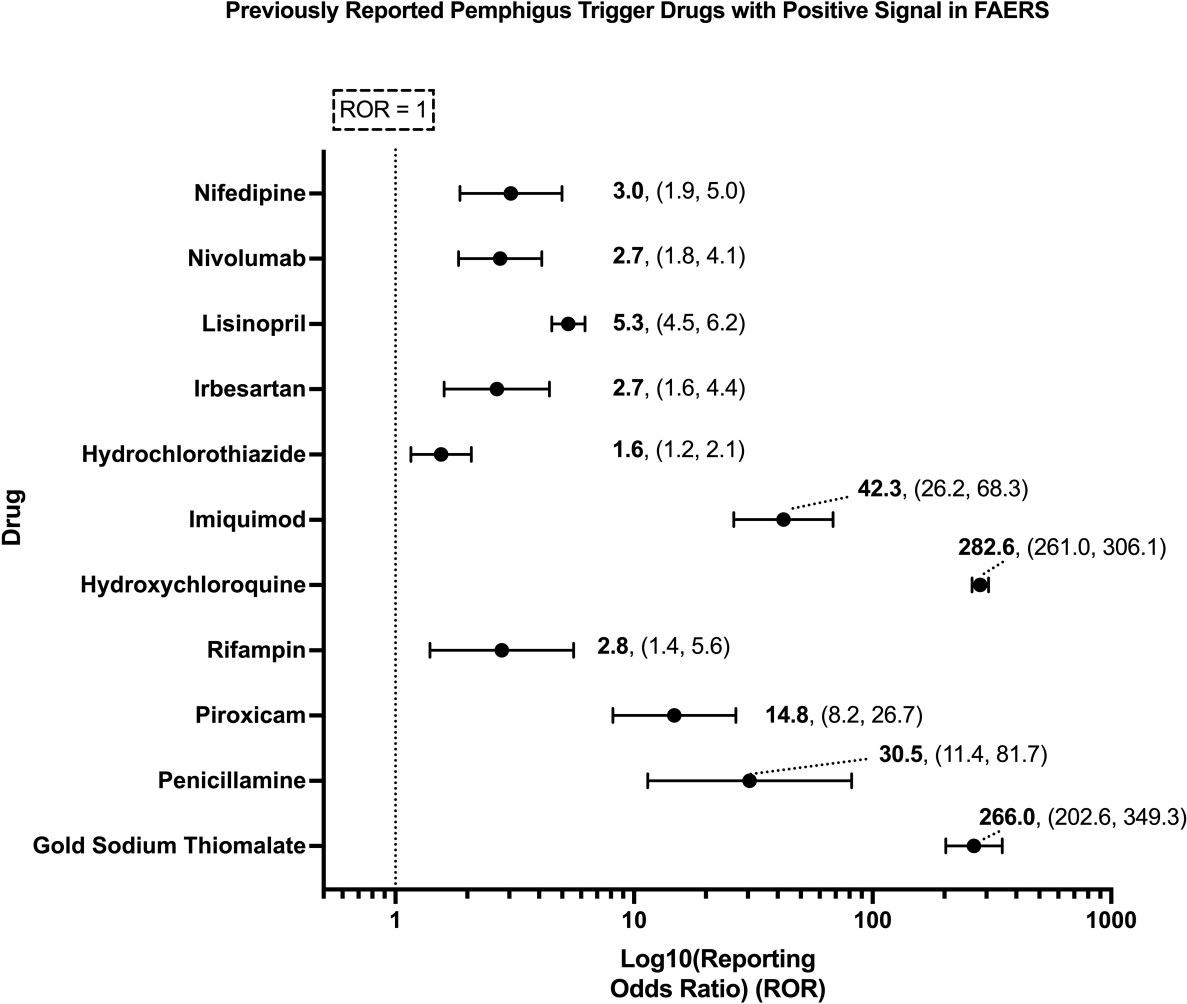
Previously reported PV trigger drugs wherein a significant pharmacovigilance signal, defined as a lower bound of the 95% CI of the ROR > 1, and χ^2^ > 4, and n > 3, was detected in the present analysis of FAERS. X-axis scale is log10.

While certain medications such as penicillamine and gold sodium thiomalate have long been associated with triggering PV across multiple decades and many case reports (23, 24, 40–44), a considerably larger number of drugs have only ever been reported to trigger PV in isolated case reports. Our analysis of spontaneously reported adverse events in FAERS was able to detect significant pharmacovigilance signals across a subset of both well-known and lesser reported putative PV trigger drugs (**Figure 2**), two of which, gold sodium thiomalate and hydroxychloroquine, had particularly high RORs (>200), while three (penicillamine, piroxicam, and imiquimod) had very strong RORs (14-45), and six, rifampin, hydrochlorothiazide, irbesartan, lisinopril, nivolumab, and nifedipine, had modestly strong RORs (2-5). While the strength of these associations, as quantified by the ROR, varied, all of the aforementioned drugs’ associations with the adverse event pemphigus satisfied the criteria for statistical significance.

### 3.4 Pharmacovigilance analysis failed to detect a significant signal among a subset of putative PV trigger drugs

**Figure 3:**
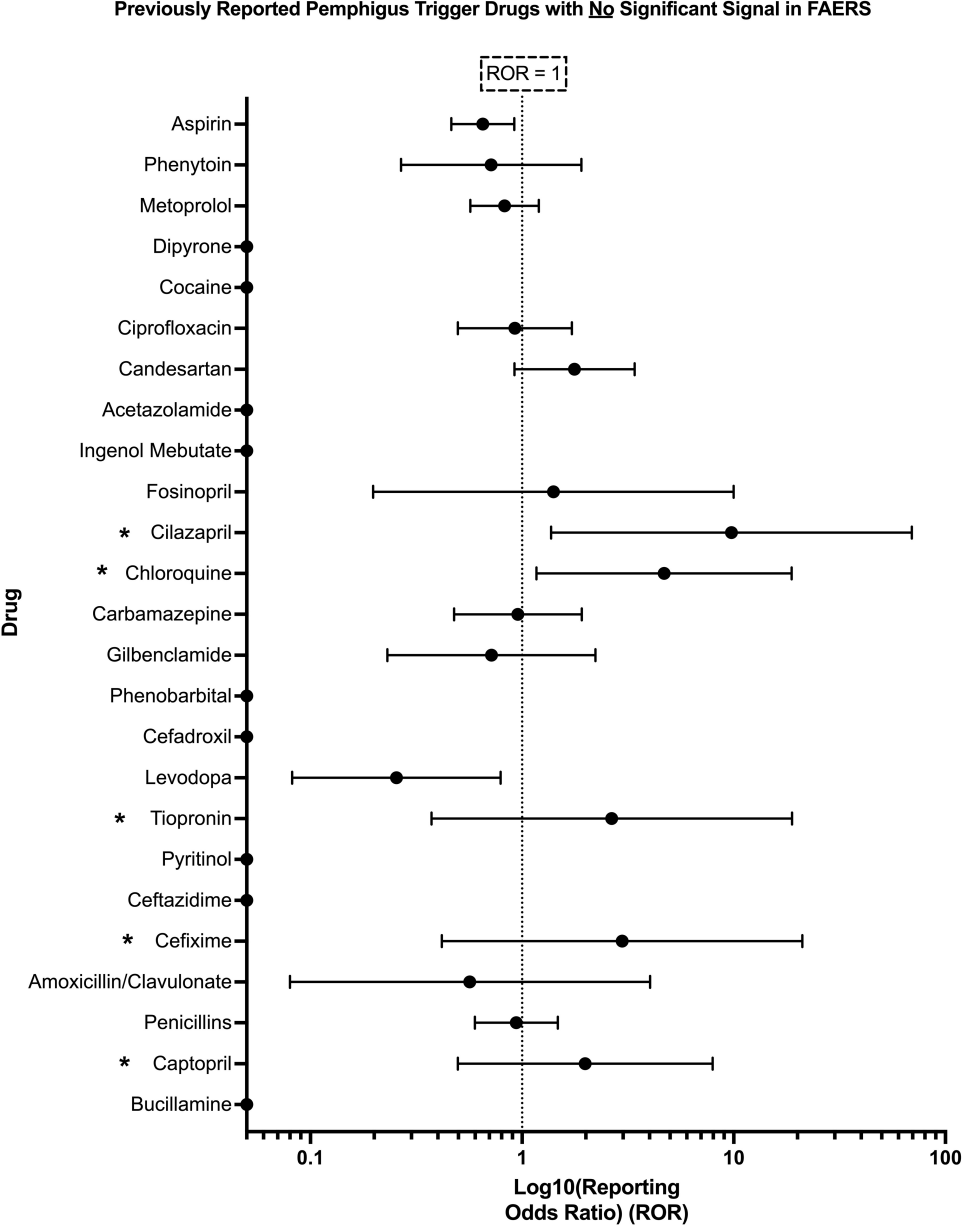
Previously reported PV trigger drugs wherein a significant pharmacovigilance signal, defined as lower bound of 95% CI of ROR > 1, and χ^2^ > 4, and n > 3, was not detected in the present analysis of FAERS. Those drugs listed with an asterisk (*) had <3 total reports in FAERS containing the combination of the given drug and the adverse event pemphigus and thus did not satisfy the criteria for significance detailed in Section 2.4. X-axis scale is log10.

The present analysis was unable to detect a significant pharmacovigilance signal for 25 out of 36 drugs previously reported in the literature to trigger PV. No significant signal was detected for: bucillamine, captopril, penicillin, amoxicillin/clavulanic acid, cefixime, ceftazidime, pyritinol, thiopronine, levodopa, aspirin, cefadroxil, phenobarbital, glibenclamide, carbamazepine, chloroquine, cilazapril, fosinopril, ingenol mebutate, acetazolamide, candesartan, ciprofloxacin, cocaine, dipyrone, metoprolol, and phenytoin (**Figure 3**).

Several drugs with RORs similar to those in the ‘significant’ set (e.g., cilazapril, chloroquine, tiopronin, cefixime, and captopril) did not meet the criteria for statistical significance due to the low number of cases reported in the FAERS database. Specifically, the number of cases for all of the aforementioned drugs was either 1 or 2 (**Supplementary Figure 1**), which falls below the previously described threshold of n > 3 required to establish a significant pharmacovigilance signal (33, 34).

## 4 Discussion

We describe a significantly increased ROR for penicillamine, gold sodium thiomalate, piroxicam, rifampin, hydroxychloroquine, imiquimod, hydrochlorothiazide, irbesartan, lisinopril, nivolumab, and nifedipine and pemphigus above that observed for all other drugs in FAERS, our comparator.

The present work has the advantage of being able to replicate the associations between a subset of the putative PV trigger drugs reported in the literature, largely across isolated case reports, in the context of a population level spontaneous reporting database with significantly larger sample sizes. While pharmacovigilance and disproportionality analysis do not establish causality, they serve to enrich the data pool available to aid clinicians and researchers in their management and study of individuals with PV. Such data is ultimately in service of the task of identifying avoidable triggers of disease and ultimately developing treatment modalities which adequately address the heterogeneous biological and clinical underpinnings and expressions of PV across individuals. While a diverse range of environmental factors such as psychosocial stressors (45), viral infections (46), and, particularly in recent years, vaccinations (47, 48), have been implicated in the onset and modification of disease in PV, our pharmacovigilance-based investigation here focuses on the critical evaluation of drugs previously linked with pemphigus.

Historically, some have proposed that “drug-*induced*” pemphigus arises primarily due to exogenous triggers, such as medications, with the disease resolving upon cessation of the offending agent, whereas “drug-*triggered*” pemphigus had been attributed to a complex interplay of endogenous, genetic, hereditary, and immunological factors, with drugs serving as secondary contributors. Previous attempts to distinguish these two etiologically drug-related subtypes of pemphigus from so-called “spontaneous” or “idiopathic” cases (49, 50) based on clinical (51) or histopathological (51) criteria have yielded inconsistent results (44), with features such as pruritus (52) and histopathological patterns (53) failing to reliably differentiate subtypes. Consistent with the views of Ruocco et al., first expressed well over a decade ago (54, 55), we propose that all cases of pemphigus likely result from the dynamic interaction of genetic predisposition with various environmental triggers, including medications, infections, stress, etc. This interplay highlights the complexity of PV’s pathogenesis, suggesting that what has been historically labeled as “spontaneous”/“idiopathic” pemphigus may, in reality, reflect unrecognized environmental factors acting on a susceptible genetic background (54), warranting further investigation into these triggers to enhance disease prevention and personalize management.

Gold sodium thiomalate, the agent found to have the highest ROR in our study, has a paradoxical relationship to pemphigus. Reports dating back to the late 1970s (40), and continuing through the early 2000s (41), document instances of pemphigus exacerbated (40) and/or triggered (42) by gold sodium thiomalate (intramuscular) therapy, as well as oral gold (auranofin) therapy (41). Conversely, the therapeutic potential of gold compounds in pemphigus has also long been recognized (56, 57). More recently, others have cautioned against its use, especially in light of the availability of newer alternatives, owing to its paradoxical ability to exacerbate the very disease it treats (54).

Antihypertensives, the largest group of drugs we found to be associated with PV, represent a therapeutic dilemma in this condition, one magnified by the prevalence of hypertension among the demographic typically affected by PV [average age of onset between 45-65 in various populations (2)] and further compounded by the chronic use of glucocorticoids, still a cornerstone of PV management. Multiple distinct classes of these drugs, including angiotensin converting enzyme (ACE) inhibitors/angiotensin receptor blockers (ARBs), calcium channel blockers (CCBs), diuretics, and beta-blockers, have been implicated in both disease induction and exacerbation in both PV (20–22, 26, 58) as well as other cutaneous diseases, as illustrated by the well-documented association of beta-blockers with psoriasis (59–61).

Of particular interest is a 2010 study of 63 hypertensive adults *without* dermatologic disease treated with ACE inhibitors where the sera of 33 (52.38%) such individuals was found to contain autoantibodies directed against an antigen of the superficial epidermis (62). Most notably, 7.8% and 6.4% of those same individuals were found to have anti-Dsg 3 and -Dsg 1 autoantibodies at values greater than or equal to 10 IU/mL by ELISA, respectively (1.5% and 3.2% with values above 20 IU/mL for Dsg1/3, respectively). In one individual, who was possibly exposed to multiple drugs previously associated with pemphigus in the literature (reported as captopril (44, 58, 63–65), as well as a beta blocker (66, 67) and a statin (68) – though the specific agents are not listed) autoantibody levels were as high as 26 IU/mL and 69 IU/mL for Dsg3/1, respectively – again, in the absence of any clinically apparent cutaneous disease. The intersection of antihypertensives and PV underscores a critical aspect of managing autoimmune diseases: the necessity of navigating the dual objectives of treating the primary condition while mitigating the risk of exacerbating it through necessary comorbid management strategies.

**Figure 4:**
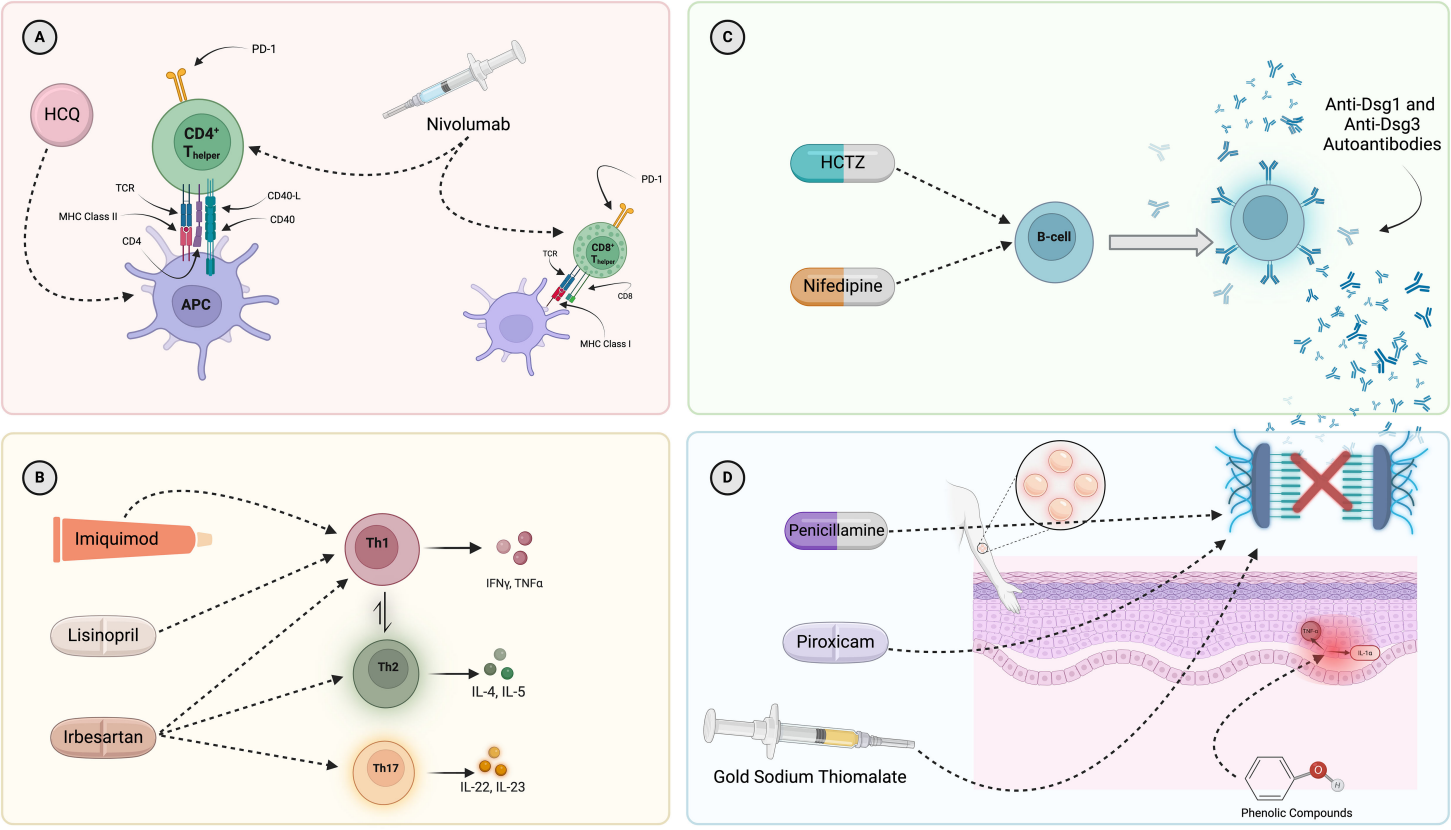
Mapping of select, known interactions of the drugs found to have a significant pharmacovigilance signal in this work on to the major immunologic drivers of PV pathogenesis. **(A)** Presentation of self-antigens (Dsg3, Dsg1, and others) via MHC Class II molecules (particularly DRB1*04:02 and DQB1*05:03) is necessary for initiation of the immune response in PV. Hydroxychloroquine and nivolumab have been reported to modulate T-cell function and antigen presentation. **(B)** A dysregulation of T-helper cell subsets, with a skewing towards Th2 and Th17, has been described in PV. Imiquimod, lisinopril, and irbesartan have been suggested to interact with various T-helper cell subsets and, subsequently, drive possible shifts in disease relevant cytokine production. **(C)** B-cells are known to produce autoantibodies in PV. Drugs known to increase B-cell numbers include hydrochlorothiazide and nifedipine. **(D)** The histopathological hallmark of PV is suprabasal acantholysis of epidermal keratinocytes. Certain drugs (penicillamine, piroxicam, gold sodium thiomalate) have been shown to be capable of biochemically inducing acantholysis in the absence of PV autoantibodies while others (phenolic compounds, such as tannic acid) have been suggested to contribute to acantholysis in PV by more indirect routes, including the induction of cytokine expression by keratinocytes and subsequent upregulation of proteinase enzymes shown to disrupt intercellular adhesion molecules. Given that rifampin is a drug which contains a phenol group, it could conceivably have a similar effect, but has not been tested directly. It must be noted that the depicted mechanisms represent selected examples of known immune pathways associated with the drugs presented and are *not* intended to provide a comprehensive assessment. They do not encompass all possible mechanisms of action for any individual drug, nor do they capture all potential immune system interactions. Most importantly, we lack sufficient evidence to conclude that the mechanisms depicted are those actually responsible for the induction or exacerbation of PV by these drugs. HCQ, hydroxychloroquine; APC, antigen-presenting cell; HCTZ, hydrochlorothiazide.

Possible mechanisms of pemphigus induction for each of the drugs found to have a positive pharmacovigilance signal in the present analysis are summarized in **Table 2**. Interestingly, all 11 of the agents found to have a positive signal in this analysis exert either direct or indirect immunomodulatory activity. Direct immunomodulators include imiquimod (an immune response modifier) and nivolumab (an immune checkpoint inhibitor). Additionally, piroxicam, penicillamine, and gold sodium thiomalate (anti-inflammatory/anti-rheumatic agents) are employed clinically for their ability to alter immune homeostasis. Hydroxychloroquine, used in the treatment of a number of inflammatory and autoimmune diseases, interacts with and alters the immune system in a myriad of ways including alteration of lysosomal acidification and inhibition of toll like receptor signaling, among others, and is discussed in greater detail elsewhere (94). In terms of indirect immunomodulation, rifampin may exert its effects via the induction of pro-inflammatory cytokine expression in keratinocytes (1, 95) and/or the downstream consequences of alterations in the microbiome. Finally, the largest single group among the drugs found to have positive pharmacovigilance signals, the anti-hypertensives, have also been shown to elicit changes in immune function, specifically: lisinopril inhibits the production of Th1 cytokines including IFN-γ and IL-12 which may facilitate an immunological shift towards Th2 dominance (96, 97), Irbesartan decreases the counts and cytokine production of Th1 and Th17 cells lines and increases the number of Th2 cells (96, 97), HCTZ promotes an increase in the number of activated B-cells, and nifedipine has been shown to reverse the uremia-associated inhibition of B-cell proliferation (96). Collectively, the drugs that are statistically linked to pemphigus share, as a unifying factor, the propensity to alter immune function, which may explain their ability to trigger the onset of disease in genetically susceptible individuals. **Figure 4** attempts to place the limited available evidence regarding potential immunologic sites of action of the drugs found to have significant associations with PV in this study in the context of known factors relevant to PV pathogenesis. Future mechanistic and translational investigations are necessary in order to examine direct impacts of specific medications on the immunome.

**Table 2:** Possible mechanisms underlying pemphigus induction by previously reported trigger drugs with a positive pharmacovigilance signal.

Drug	PV Trigger Drug Grouping	Summary of Previous Evidence in Pemphigus	Other Clinically Significant Associations
Gold Sodium Thiomalate	Thiols	Implicated in both triggering (41, 42) and exacerbating (40) pemphigus. Paradoxically also used as treatment for pemphigus in the past (56, 57, 69). See discussion for more detail.	Used in treatment of rheumatoid arthritis which has been shown by this group to cluster with pemphigus (9).
Penicillamine	Thiols	First agent implicated in drug induced pemphigus (23). An early study showed that approximately 7% of individuals taking penicillamine for > 6 months developed pemphigus (24). Has been shown to be able to produce acantholysis *in vitro* in the absence of pemphigus autoantibodies (43, 70).	Has also been reported to trigger other autoimmune diseases including systemic lupus erythematosus and myasthenia gravis, leading some to suggest that penicillamine produces a dysregulation of the immune system that manifests as an increased tendency towards autoantibody production (44, 71).
Piroxicam	Thiols	Tends to produce suprabasal acantholysis *in vitro* whereas subcorneal acantholysis is more commonly seen clinically in cases of piroxicam induced pemphigus (70, 72), though both are possible (73).	Has also been reported to trigger a number of other cutaneous adverse events including: linear IgA bullous dermatosis (74), exfoliative dermatitis, and lupus erythematosus, among others (74).
Rifampin (=Rifampicin)	Phenols	Has been reported to both exacerbate (75, 76) pemphigus as well as trigger it *de novo (* 77). It has been suggested that rifampin contributes to flares of existing pemphigus by altering the metabolism of prednisone and other glucocorticoids, for example by induction of CYP3A4 leading to a decline of 30-60% of those drugs’ area under concentration-time curve. Has also been shown to interact with metabolism of mycophenolate by altering glucuronidation (75).	Some have suggested increasing the dose of prednisone by 2-3x upon initiation of concomitant treatment with rifampin in cases where neither can safely be avoided or discontinued (such as a pemphigus patient with active disease who develops pulmonary tuberculosis) (75).
Hydroxychloroquine	Non-Thiol, Non-Phenols	Previously reported to trigger pemphigus in an isolated case report (27); recently, this group has reported a positive pharmacovigilance signal between exposure to hydroxychloroquine and reporting of the adverse event pemphigus to the FDA at the population level via disproportionality analysis of the FDA Adverse Event Reporting System (FAERS) (78).	Cutaneous adverse events related to hydroxychloroquine exposure are common and range in severity from relatively mild (e.g. drug eruption/rash, its most commonly reported cutaneous adverse event, or cutaneous hyperpigmentation) (79) to potentially life threatening (such as acute generalized exanthematous pustulosis (79, 80), Stevens Johnson Syndrome, and Toxic Epidermal Necrolysis) (79).
Imiquimod	Non-Thiol, Non-Phenols	Associated with both the induction (81–85) as well as exacerbation (86) of various forms of pemphigus. Suggested to act in pemphigus by increasing the production of multiple cytokines: IFN-⍺, TNF-⍺, IL-1, IL-1 receptor antagonist, IL-6, IL-8, IL-10, IL-12 (81, 87). Many of the aforementioned cytokines overlap with those shown to be elevated in the sera of patients with pemphigus. Additionally, therapeutic exposure to IFN-⍺ has been reported to trigger pemphigus (88, 89) as well as other cutaneous diseases (90).	Has also been reported to trigger psoriasis, vitiligo, as well as a flare of myasthenia gravis (86). One study of individuals with either malignant melanoma or T-cell lymphoma and treated with IFN-⍺ reported the induction of auto antibodies against epidermal antigens (including intracellular substance and/or basement membrane) after 6 months of treatment in 32% (15/47) of patients, notably in the absence of any clinically apparent cutaneous disease (91).
Hydrochlorothiazide	Non-Thiol, Non-Phenols	See Discussion for review of the potential mechanisms of pemphigus induction by anti-hypertensive agents of various classes^a^.	
Irbesartan	Non-Thiol, Non-Phenols		
Lisinopril	Non-Thiol, Non-Phenols		
Nifedipine	Non-Thiol, Non-Phenols		
Nivolumab	Other - Immunotherapy	Immune related adverse events (irAEs) are common among those treated with immune checkpoint inhibitors such as nivolumab. 42-66% of irAEs are cutaneous, ranging in severity from mild to potentially life threatening, e.g. pemphigus. Mechanisms of irAEs are poorly understood though thought to be T-cell mediated (92).	Reporting of novel autoimmune adverse events and/or flares of pre-existing, quiescent autoimmune diseases among individuals exposed to immune checkpoint inhibitors is essential as patients with pre-existing autoimmune diseases were excluded from most clinical trials of these drugs (92).

a While beta blockers are no longer used as first line anti-hypertensive agents, they remain in use in select cases, particularly in the presence of certain co-morbid conditions such as angina pectoris, hyperthyroidism, or migraine, among others (93).

While the drugs with significant pharmacovigilance signals identified in this work are unified broadly by a capacity for immunomodulation, those drugs are, of course, not the only agents capable of exerting such effects on immune function. For the overwhelming majority of PV-associated drugs reported in the literature there is a lack of substantive experimental evidence regarding the potential immune mechanisms at play. Where evidence is available, a diverse range of pathways including biochemical drug-self-antigen interactions resulting in neoantigen formation and subsequent immune activation as well as drug induced alterations of cytokine expression patterns have variously been implicated (1, 20–22, 63, 95, 98, 99) (see **Figure 1** and **Table 2**) as potential contributors to the breakdown of immune tolerance that is the *sine qua non* of all autoimmune phenomena.

We note that the temporal relationship between drug exposure and disease onset varies widely in the literature. For instance, one study reported an average latency of 154.27 days between drug exposure and PV onset, with ranges of 172–1140 days depending on the specific drug involved, though this study only reported data on a small fraction of all previously reported putative PV trigger drugs (21). Other reports suggest that environmental exposures occurring years, and in some cases decades (15 years (100), 53 years (49), etc.), prior to symptom onset may also contribute to disease development (49, 100–102). These findings underscore the complexity of PV pathogenesis, where both proximal (<1 year) and remote (>1 year) exposures may act as triggers, likely in the context of cumulative immune dysregulation over time.

Despite the lack of definitive mechanistic explanations linking exposure to the drugs examined here to the development of clinically significant disease, the strong statistical evidence for the association between exposure to the subset of drugs validated in this work and the development of pemphigus supports our hypothesis, and corroborates previous case report-based evidence, that those drugs likely represent trigger factors for the development of pemphigus. It must be noted that, insofar as the subset of drugs which did not produce significant pharmacovigilance signals, i.e. those that were not validated by the present approach, are concerned this work does *not* rule out the possibility that some or all of those agents may well represent bona fide triggers of PV which simply failed to be detected by our present pharmacovigilance-based approach. Given the severity of PV, the burden associated with both the disease itself and its still primarily glucocorticoid based treatment, and the outsized role of drugs in triggering PV as evidenced by previous systematic reviews, future studies capable of establishing causality are warranted for all reasonably suspect putative trigger drugs.

We acknowledge several limitations of this observational, retrospective, pharmacovigilance analysis. Reports in FAERS are not FDA-validated prior to database entry, and the quality of individual reports can be inconsistent, with some missing details about dosage, demographics, or the adverse event itself (30, 103). A given adverse event reported in FAERS cannot be definitively attributed to any particular drug exposure at the level of the individual (103). Sakaeda et al. highlighted the fact that while individual reports may be anecdotal, in aggregate, they can reflect a more accurate picture, writing that “[one] report in the FAERS database is a story, sometimes only a rumor, but numerous reports can reflect reality” (104). An additional limitation of this study is the potential underrepresentation of drugs that did not show significant associations with pemphigus in our analysis. The absence of a significant signal for certain drugs, despite reported associations in the literature, may reflect factors such as reporting bias, underreporting, or variability in prescription frequency rather than a true absence of etiological significance in PV. Ultimately, this pharmacovigilance disproportionality research serves as a foundation for hypothesis generation for future work on establishing conclusive evidence of causality.

## Data Availability

Publicly available datasets were analyzed in this study. This data can be found here: https://openvigil.sourceforge.net/.

## References

[1] Brenner S Mashiah J Tamir E Goldberg I Wohl Y. PEMPHIGUS: an acronym for a disease with multiple etiologies. SKINmed: Dermatol Clinician. (2003) 2:163–7. doi: 10.1111/j.1540-9740.2003.02040.x 14673292

[2] Schmidt E Kasperkiewicz M Joly P. Pemphigus. Lancet. (2019) 394:882–94. doi: 10.1016/S0140-6736(19)31778-7 31498102

[3] Seiffert-Sinha K Khan S Attwood K Gerlach JA Sinha AA. Anti-thyroid peroxidase reactivity is heightened in pemphigus vulgaris and is driven by human leukocyte antigen status and the absence of desmoglein reactivity. Front Immunol. (2018) 9:625. doi: 10.3389/fimmu.2018.00625 29675021 PMC5896579

[4] Sinha AA Sajda T. The evolving story of autoantibodies in pemphigus vulgaris: development of the “Super compensation hypothesis. Front Med (Lausanne). (2018) 5:218. doi: 10.3389/fmed.2018.00218 30155465 PMC6102394

[5] Nguyen VT Ndoye A Grando SA. Pemphigus vulgaris antibody identifies pemphaxin. A novel keratinocyte annexin-like molecule binding acetylcholine. J Biol Chem. (2000) 275:29466–76. doi: 10.1074/jbc.M003174200 10899159

[6] Ahmed AR Carrozzo M Caux F Cirillo N Dmochowski M Alonso AE. Monopathogenic vs multipathogenic explanations of pemphigus pathophysiology. Exp Dermatol. (2016) 25:839–46. doi: 10.1111/exd.13106 27305362

[7] Kalantari-Dehaghi M Anhalt GJ Camilleri MJ Chernyavsky AI Chun S Felgner PL. Pemphigus vulgaris autoantibody profiling by proteomic technique. PloS One. (2013) 8:e57587. doi: 10.1371/journal.pone.0057587 23505434 PMC3591405

[8] Rosi-Schumacher M Baker J Waris J Seiffert-Sinha K Sinha AA. Worldwide epidemiologic factors in pemphigus vulgaris and bullous pemphigoid. Front Immunol. (2023) 14:1159351. doi: 10.3389/fimmu.2023.1159351 37180132 PMC10166872

[9] Parameswaran A Attwood K Sato R Seiffert-Sinha K Sinha AA. Identification of a new disease cluster of pemphigus vulgaris with autoimmune thyroid disease, rheumatoid arthritis and type I diabetes. Br J Dermatol. (2015) 172:729–38. doi: 10.1111/bjd.13433 25272088

[10] Baker J Seiffert-Sinha K Sinha AA. Patient genetics shape the autoimmune response in the blistering skin disease pemphigus vulgaris. Front Immunol. (2023) 13:1064073. doi: 10.3389/fimmu.2022.1064073 36703961 PMC9871500

[11] Sinha AA. The genetics of pemphigus. Dermatol Clin. (2011) 29(3):381–vii. doi: 10.1016/j.det.2011.03.020 21605803

[12] Sinha AA Brautbar C Szafer F Friedmann A Tzfoni E Todd JA. A newly characterized HLA DQβ Allele associated with pemphigus vulgaris. Science. (1988) 239:1026–9. doi: 10.1126/science.2894075 2894075

[13] Todd JA Acha-Orbea H Bell JI Chao N Fronek Z Jacob CO. A molecular basis for MHC class II—Associated autoimmunity. Science. (1988) 240:1003–9. doi: 10.1126/science.3368786 3368786

[14] Sinha AA Lopez MT McDevitt HO. Autoimmune diseases: the failure of self tolerance. Science. (1990) 248:1380–8. doi: 10.1126/science.1972595 1972595

[15] Salathiel AM BroChado MJF Kim O Deghaide NHS Donadi EA Roselino AM. Family study of monozygotic twins affected by pemphigus vulgaris. Hum Immunol. (2016) 77:600–4. doi: 10.1016/j.humimm.2016.05.005 27177496

[16] Selmi C Lu Q Humble MC. Heritability versus the role of the environment in autoimmunity. J Autoimmun. (2012) 39:249–52. doi: 10.1016/j.jaut.2012.07.011 22980030

[17] Somers EC Thomas SL Smeeth L Hall AJ. Autoimmune Diseases Co-Occurring within Individuals and within Families: A Systematic Review. Epidemiology. (2006) 17:202–17. doi: 10.1097/01.ede.0000193605.93416.df 16477262

[18] Mohr DC Hart SL Julian L Cox D Pelletier D. Association between stressful life events and exacerbation in multiple sclerosis: a meta-analysis. BMJ. (2004) 328:731. doi: 10.1136/bmj.38041.724421.55 15033880 PMC381319

[19] Wild CP. Complementing the genome with an “Exposome”: the outstanding challenge of environmental exposure measurement in molecular epidemiology. Cancer Epidemiology Biomarkers Prev. (2005) 14:1847–50. doi: 10.1158/1055-9965.EPI-05-0456 16103423

[20] Tavakolpour S. Pemphigus trigger factors: special focus on pemphigus vulgaris and pemphigus foliaceus. Arch Dermatol Res. (2018) 310:95–106. doi: 10.1007/s00403-017-1790-8 29110080

[21] Ghaedi F Etesami I Aryanian Z Kalantari Y Goodarzi A Teymourpour A. Drug-induced pemphigus: A systematic review of 170 patients. Int Immunopharmacology. (2021) 92:107299. doi: 10.1016/j.intimp.2020.107299 33418246

[22] Adebiyi OT Galloway DF Augustin MS Sinha AA. The multifactorial complexities of autoimmune development in Pemphigus vulgaris: Critical evaluation of the role of environmental and lifestyle “exposome” factors. Front Immunol. (2022) 13:1058759. doi: 10.3389/fimmu.2022.1058759 36703956 PMC9871583

[23] Degos R Touraine R Belaïch S Revuz J. Pemphigus in a patient treated with penicillamine for Wilson’s disease. Bull Soc Fr Dermatol Syphiligr. (1969) 76:751–3. 5384189

[24] Marsden RA Vanhegan RI Walshe M Hill H Mowat AG. Pemphigus foliaceus induced by penicillamine. Br Med J. (1976) 2:1423–4. doi: 10.1136/bmj.2.6049.1423 PMC1690441 1009359

[25] Goldberg I Ingher A Brenner S. Pemphigus vulgaris triggered by rifampin and emotional stress. Skinmed. (2004) 3:294. doi: 10.1111/j.1540-9740.2004.03343.x 15365272

[26] Kim SC Won JH Ahn SK. Pemphigus foliaceus induced by nifedipine. Acta Derm Venereol. (1993) 73:210–1. doi: 10.2340/000155555573210211 8105622

[27] Ghaffarpour G Jalali MHA Yaghmaii B Mazloomi S Soltani-Arabshahi R. Chloroquine/hydroxychloroquine-induced pemphigus. Int J Dermatol. (2006) 45:1261–3. doi: 10.1111/j.1365-4632.2006.03075.x 17040465

[28] Kim MK Lee SK. Drug-induced pemphigus-like lesion accompanied by severe gingival enlargement. Oral Biol Res. (2020) 44:140–7. doi: 10.21851/obr.44.04.202012.140

[29] Guo Q Duan S Liu Y Yuan Y. Adverse drug events in the prevention and treatment of COVID-19: A data mining study on the FDA adverse event reporting system. Front Pharmacol. (2022) 13:2023. doi: 10.3389/fphar.2022.954359 PMC9730807 36506542

[30] Center for Drug Evaluation and Research. FDA adverse event reporting system (FAERS) public dashboard . FDA. Available online at: https://www.fda.gov/drugs/questions-and-answers-fdas-adverse-event-reporting-system-faers/fda-adverse-event-reporting-system-faers-public-dashboard (Accessed October 12, 2023).

[31] Anatomical therapeutic chemical (ATC) classification. Available online at: https://www.who.int/tools/atc-ddd-toolkit/atc-classification (Accessed February 2, 2024).

[32] Singh AP Tousif S Umbarkar P Lal H. A pharmacovigilance study of hydroxychloroquine cardiac safety profile: potential implication in COVID-19 mitigation. J Clin Med. (2020) 9:1867. doi: 10.3390/jcm9061867 32549293 PMC7355808

[33] Böhm R. Primer on disproportionality analysis. (2018).

[34] Böhm R von Hehn L Herdegen T Klein H-J Bruhn O Petri H. OpenVigil FDA - inspection of U.S. American adverse drug events pharmacovigilance data and novel clinical applications. PloS One. (2016) 11:e0157753. doi: 10.1371/journal.pone.0157753 27326858 PMC4915658

[35] Evans SJW Waller PC Davis S. Use of proportional reporting ratios (PRRs) for signal generation from spontaneous adverse drug reaction reports. Pharmacoepidemiology Drug Safety. (2001) 10:483–6. doi: 10.1002/pds.677 11828828

[36] Tian X Zheng S Wang J Yu M Lin Z Qin M. Cardiac disorder-related adverse events for aryl hydrocarbon receptor agonists: a safety review. Expert Opin Drug Saf. (2022) 21:1505–10. doi: 10.1080/14740338.2022.2078301 35582860

[37] Javed F Kumar A. Identification of signal of clindamycin associated renal failure acute: A disproportionality analysis. Curr Drug Saf. (2024) 19:123–8. doi: 10.2174/1574886318666230228142856 36852785

[38] Böhm R Bulin C Waetzig V Cascorbi I Klein HJ Herdegen T. Pharmacovigilance-based drug repurposing: The search for inverse signals via OpenVigil identifies putative drugs against viral respiratory infections. Br J Clin Pharmacol. (2021) 87:4421–31. doi: 10.1111/bcp.14868 33871897

[39] van Puijenbroek EP Bate A Leufkens HGM Lindquist M Orre R Egberts ACG. A comparison of measures of disproportionality for signal detection in spontaneous reporting systems for adverse drug reactions. Pharmacoepidemiol Drug Saf. (2002) 11:3–10. doi: 10.1002/pds.668 11998548

[40] Miyamoto Y Maeda M. Pemphigus induced by gold sodium thiomalate. Arch Dermatol. (1978) 114:1855. doi: 10.1001/archderm.1978.01640240079030 104664

[41] Lo Schiavo A Sangiuliano S Puca RV Brunetti G Ruocco E Cozzi R. Pemphigus and chrysotherapy: all that glitters is not gold! Int J Dermatol. (2008) 47:645–7. doi: 10.1111/j.1365-4632.2008.03530.x 18477174

[42] Ciompi ML Marchetti G Bazzichi L Puccetti L Agelli M. D-penicillamine and gold salt treatments were complicated by myasthenia and pemphigus, respectively, in the same patient with rheumatoid arthritis. Rheumatol Int. (1995) 15:95–7. doi: 10.1007/BF00302124 8588126

[43] Zone J Ward J Boyce E Schupbach C. Penicillamine-induced pemphigus. JAMA. (1982) 247:2705–7. doi: 10.1001/jama.1982.03320440053036 7077766

[44] Korman NJ Eyre RW Zone J Stanley JR. Drug-induced pemphigus: autoantibodies directed against the pemphigus antigen complexes are present in penicillamine and captopril-induced pemphigus. J Invest Dermatol. (1991) 96:273–6. doi: 10.1111/1523-1747.ep12464471 1991988

[45] Davis AE Nathanson J Attwood K Sinha AA Seiffert-Sinha K. A retrospective analysis of patient-reported physical and psychological stressors as trigger factors in autoimmune bullous disease. Arch Dermatol Res. (2024) 316:515. doi: 10.1007/s00403-024-03240-5 39133440

[46] Baroukhian J Seiffert-Sinha K Sinha AA. Response to Kasperkiewicz et al.’s “Pemphigus following herpes simplex infection: A global comprehensive cohort study. J Am Acad Dermatol. (2024) 0. doi: 10.1016/j.jaad.2024.07.1536 39579998

[47] Moro F Sinagra JLM Salemme A Fania L Mariotti F Pira A. Pemphigus: trigger and predisposing factors. Front Med (Lausanne). (2023) 10:1326359. doi: 10.3389/fmed.2023.1326359 38213911 PMC10783816

[48] Corrá A Barei F Genovese G Zussino M Spigariolo CB Mariotti EB. Five cases of new-onset pemphigus following vaccinations against coronavirus disease 2019. J Dermatol. (2023) 50:229–33. doi: 10.1111/1346-8138.16554 PMC9538601 35975548

[49] Ruocco V Pisani M. Induced pemphigus. Arch Dermatol Res. (1982) 274:123–40. doi: 10.1007/BF00510366 7165361

[50] Wolf R Tamir A Brenner S. Drug-induced versus drug-triggered pemphigus. Dermatologica. (1991) 182:207–10. doi: 10.1159/000247795 1884854

[51] Kardaun SH de Sena Nogueira Maehara L. Drug-induced pemphigus. In: Horváth B, editor. Autoimmune bullous diseases: text and review. Cham: Springer International Publishing (2022). p. 99–102. doi: 10.1007/978-3-030-91557-5_12

[52] Rolader R Daugherty LN Liu Y Feldman RJ. Prevalence and predictors of pruritus in pemphigus compared with bullous pemphigoid: A cross-sectional study. J Am Acad Dermatol. (2020) 83:251–4. doi: 10.1016/j.jaad.2020.01.025 31962093

[53] Landau M Brenner S. Histopathologic findings in drug-induced pemphigus. Am J Dermatopathol. (1997) 19:411–4. doi: 10.1097/00000372-199708000-00017 9261480

[54] Ruocco V Ruocco E Lo Schiavo A Brunetti G Guerrera LP Wolf R. Pemphigus: Etiology, pathogenesis, and inducing or triggering factors: Facts and controversies. Clinics Dermatol. (2013) 31:374–81. doi: 10.1016/j.clindermatol.2013.01.004 23806154

[55] Brenner S Wolf R Ruocco V. Contact pemphigus: a subgroup of induced pemphigus. Int J Dermatol. (1994) 33:843–5. doi: 10.1111/j.1365-4362.1994.tb01016.x 7883405

[56] Penneys NS Eaglstein WH Indgin S Frost P. Gold sodium thiomalate treatment of pemphigus. Arch Dermatol. (1973) 108:56–60. doi: 10.1001/archderm.1973.01620220028007 4197772

[57] Penneys NS Eaglstein WH Frost P. Management of pemphigus with gold compounds: A long-term follow-up report. Arch Dermatol. (1976) 112:185–7. doi: 10.1001/archderm.1976.01630260013004 822783

[58] Pietkiewicz P Gornowicz-Porowska J Bowszyc-Dmochowska M Dmochowski M. A retrospective study of antihypertensives in pemphigus: a still unchartered odyssey particularly between thiols, amides and phenols. Arch Med Sci. (2015) 11:1021–7. doi: 10.5114/aoms.2015.54857 PMC4624747 26528346

[59] Awad VM Sakhamuru S Kambampati S Wasim S Malik BH. Mechanisms of Beta-Blocker Induced Psoriasis, and Psoriasis De Novo at the Cellular Level. Cureus. (2020) 12(7):e8964. doi: 10.7759/cureus.8964 32766006 PMC7398737

[60] Gold MH Holy AK Roenigk HH. Beta-blocking drugs and psoriasis: A review of cutaneous side effects and retrospective analysis of their effects on psoriasis. J Am Acad Dermatol. (1988) 19:837–41. doi: 10.1016/S0190-9622(88)70242-X 2903871

[61] Azzouz B De Guizelin A Lambert A Fresse A Morel A Trenque T. Psoriasis risk after beta-blocker exposure: Description of a pharmacovigilance signal. Br J Clin Pharmacol. (2022) 88:3813–8. doi: 10.1111/bcp.15330 35352377

[62] Cozzani E Rosa GM Drosera M Intra C Barsotti A Parodi A. ACE inhibitors can induce circulating antibodies directed to antigens of the superficial epidermal cells. Arch Dermatol Res. (2011) 303:327–32. doi: 10.1007/s00403-010-1060-5 20563876

[63] Kitamura K Aihara M Osawa J Naito S Ikezawa Z. Sulfhydryl drug-induced eruption: a clinical and histological study. J Dermatol. (1990) 17:44–51. doi: 10.1111/j.1346-8138.1990.tb01608.x 2139441

[64] Kuechle MK Hutton KP Muller SA. Angiotensin-converting enzyme inhibitor-induced pemphigus: three case reports and literature review. Mayo Clinic Proc. (1994) 69:1166–71. doi: 10.1016/S0025-6196(12)65770-X 7967779

[65] Parfrey PS Clement M Vandenburg MJ Wright P. Captopril-induced pemphigus. Br Med J. (1980) 281:194. doi: 10.1136/bmj.281.6234.194 PMC1713631 6996785

[66] Patel S Kim S Allen C. Metoprolol-induced pemphigus-like reaction. Clin Adv Periodontics. (2019) 9:24–8. doi: 10.1002/cap.10044 31490034

[67] Tomer L Ramos M Rozycki GF Hultman CS Mohammed A. The first reported case of metoprolol-induced pemphigus foliaceus in the United States: A critical report and review of literature. Cureus. (2020) 12(7):e9203. doi: 10.7759/cureus.9203 32821557 PMC7429674

[68] Lo Schiavo A Puca RV Romano F Cozzi R. Pemphigus erythematosus relapse associated with atorvastatin intake. Drug Design Dev Ther. (2014) 8:1463–5. doi: 10.2147/DDDT.S66666 PMC4173814 25258514

[69] Poulin Y Perry HO Muller SA. Pemphigus vulgaris: Results of treatment with gold as a steroid-sparing agent in a series of thirteen patients. J Am Acad Dermatol. (1984) 11:851–7. doi: 10.1016/S0190-9622(84)80463-6 6439764

[70] De Dobbeleer G Godfrine S Gourdain JM De Graef C Heenen M. *In vitro* acantholysis induced by D-penicillamine, captopril, and piroxicam on dead de-epidermized dermis. J Cutan Pathol. (1992) 19:181–6. doi: 10.1111/j.1600-0560.1992.tb01656.x 1401343

[71] Nagao K Tanikawa A Yamamoto N Amagai M. Decline of anti-desmoglein 1 IgG ELISA scores by withdrawal of D-penicillamine in drug-induced pemphigus foliaceus. Clin Exp Dermatol. (2005) 30:43–5. doi: 10.1111/j.1365-2230.2004.01655.x 15663502

[72] Piette-Brion B de Bast C Chamoun E de Dobbeleer G André J Huybrechts A. Superficial pemphigus during the treatment of rheumatoid polyarthritis with D-penicillamine and piroxicam (Feldene). Dermatologica. (1985) 170:297–301. 4018339

[73] Martin RL McSweeney GW Schneider J. Fatal pemphigus vulgaris in a patient taking piroxicam. N Engl J Med. (1983) 309:795–6. doi: 10.1056/NEJM198309293091315 6888461

[74] Camilleri M Pace JL. Linear IgA bullous dermatosis induced by piroxicam. J Eur Acad Dermatol Venereology. (1998) 10:70–2. doi: 10.1016/S0926-9959(97)00112-8 9552762

[75] Hu L Sun Y Gao Z Wang P. Pemphigus vulgaris aggravated: rifampicin found at the scene of the crime. Cutis. (2022) 109:E19–21. doi: 10.12788/cutis.0526 35856757

[76] Osipowicz K Kowalewski C Woźniak K. Mycobacterium tuberculosis and pemphigus vulgaris. Postepy Dermatol Alergol. (2018) 35:532–4. doi: 10.5114/ada.2018.72744 PMC6232536 30429716

[77] Gange RW Rhodes EL Edwards CO Powell MEA. Pemphigus induced by rifampicin. Br J Dermatol. (1976) 95:445–8. doi: 10.1111/j.1365-2133.1976.tb00849.x 974031

[78] Baroukhian J Seiffert-Sinha K Attwood K Sinha AA. Evaluation of link between COVID-19 adjacent spike in hydroxychloroquine use and increased reports of pemphigus: a disproportionality analysis of the FDA Adverse Event Reporting System. Front Immunol. (2024) 15:1470660. doi: 10.3389/fimmu.2024.1470660 39759530 PMC11695399

[79] Sharma AN Mesinkovska NA Paravar T. Characterizing the adverse dermatologic effects of hydroxychloroquine: A systematic review. J Am Acad Dermatol. (2020) 83:563–78. doi: 10.1016/j.jaad.2020.04.024 32289395

[80] Delaleu J Deniau B Battistella M de Masson A Bensaid B Jachiet M. Acute generalized exanthematous pustulosis induced by hydroxychloroquine prescribed for COVID-19. J Allergy Clin Immunol Pract. (2020) 8:2777–2779.e1. doi: 10.1016/j.jaip.2020.05.046 32525093 PMC7276124

[81] Mashiah J Brenner S. Possible mechanisms in the induction of pemphigus foliaceus by topical imiquimod treatment. Arch Dermatol. (2005) 141:908–9. doi: 10.1001/archderm.141.7.908 16027316

[82] Bauza A Del Pozo LJ Saus C Martin A. Pemphigus-like lesions induced by imiquimod. Clin Exp Dermatol. (2009) 34:e60–62. doi: 10.1111/j.1365-2230.2008.03181.x 19438577

[83] Campagne G Roca M Martínez A. Successful treatment of a high-grade intraepithelial neoplasia with imiquimod, with vulvar pemphigus as a side effect. Eur J Obstetrics Gynecology Reprod Biol. (2003) 109:224–7. doi: 10.1016/S0301-2115(02)00482-7 12860347

[84] Zhong CS Hasbun MT Jones KM Schmidt BAR Hussain SH. Pemphigus-like eruption as a complication of molluscum contagiosum treatment with imiquimod in a 5-year-old girl. Pediatr Dermatol. (2020) 37:379–80. doi: 10.1111/pde.14115 32027759

[85] Lin R Ladd J Dan J Powell DJ Way BV. Localized pemphigus foliaceus induced by topical imiquimod treatment. Arch Dermatol. (2004) 140:889–90. doi: 10.1001/archderm.140.7.889 15262711

[86] Sebaratnam DF Martin LK Rubin AI Tran K Pas HH Marr PJ. Reversible relapse of pemphigus foliaceus triggered by topical imiquimod suggests that Toll-like receptor 7 inhibitors may be useful treatments for pemphigus. Clin Exp Dermatol. (2011) 36:91–3. doi: 10.1111/j.1365-2230.2010.03918.x 20819088

[87] Farid MR de A Lugão HB Julio TA Donadi EA Bueno Filho R Roselino AM. Imiquimod-associated pemphigus foliaceus. Am J Ther. (2022) 29:e716. doi: 10.1097/MJT.0000000000001251 33021541

[88] Niizeki H Inamoto N Nakamura K Tsuchimoto K Hashimoto T Nishikawa T. A case of pemphigus foliaceus after interferon alpha-2a therapy. Dermatology. (1994) 189 Suppl 1:129–30. doi: 10.1159/000246954 8049554

[89] Marinho RT Johnson NW Fatela NM Narcisa M Serejo FS Gloria H. Oropharyngeal pemphigus in a patient with chronic hepatitis C during interferon alpha-2a therapy. Eur J Gastroenterol Hepatology. (2001) 13:869. doi: 10.1097/00042737-200107000-00017 11474319

[90] Afshar M Martinez AD Gallo RL Hata TR. Induction and exacerbation of psoriasis with Interferon-alpha therapy for hepatitis C: a review and analysis of 36 cases. J Eur Acad Dermatol Venereol. (2013) 27:771–8. doi: 10.1111/j.1468-3083.2012.04582.x PMC3443510 22671985

[91] Fleischmann M Célérier P Bernard P Litoux P Dreno B. Long-term interferon-alpha therapy induces autoantibodies against epidermis. Dermatology. (2009) 192:50–5. doi: 10.1159/000246315 8832953

[92] Krammer S Krammer C Salzer S Bağci IS French LE Hartmann D. Recurrence of pemphigus vulgaris under nivolumab therapy(2019) (Accessed February 12, 202). 10.3389/fmed.2019.00262 PMC6861207 31781569

[93] Considerations for individualizing antihypertensive therapy - UpToDate. UpToDate. Available online at: https://www.uptodate.com/contents/search?search=hypertension%20treatment&sp=0&searchType=PLAIN_TEXT&source=USER_INPUT&searchControl=TOP_PULLDOWN&searchOffset=1&autoComplete=true&language=&max=0&index=1~10&autoCompleteTerm=hypertension&rawSentence (Accessed February 6, 2024).

[94] Niemann B Puleo A Stout C Markel J Boone BA. Biologic functions of hydroxychloroquine in disease: from COVID-19 to cancer. Pharmaceutics. (2022) 14:2551. doi: 10.3390/pharmaceutics14122551 36559044 PMC9787624

[95] Brenner S Goldberg I. Drug-induced pemphigus. Clin Dermatol. (2011) 29:455–7. doi: 10.1016/j.clindermatol.2011.01.016 21679874

[96] Felkle D Jarczyński M Kaleta K Zięba K Nazimek K. The immunomodulatory effects of antihypertensive therapy: A review. Biomed Pharmacother. (2022) 153:113287. doi: 10.1016/j.biopha.2022.113287 35728352

[97] Bryniarski P Nazimek K Marcinkiewicz J. Immunomodulatory activity of the most commonly used antihypertensive drugs—Angiotensin converting enzyme inhibitors and angiotensin II receptor blockers. Int J Mol Sci. (2022) 23:1772. doi: 10.3390/ijms23031772 35163696 PMC8836033

[98] Goldberg I Kashman Y Brenner S. The induction of pemphigus by phenol drugs. Int J Dermatol. (1999) 38:888–92. doi: 10.1046/j.1365-4362.1999.00836.x 10632765

[99] Newby CS Barr RM Greaves MW Mallet AI. Cytokine release and cytotoxicity in human keratinocytes and fibroblasts induced by phenols and sodium dodecyl sulfate. J Invest Dermatol. (2000) 115:292–8. doi: 10.1046/j.1523-1747.2000.00056.x 10951249

[100] Matz H Bialy-Golan A Brenner S. Diclofenac: a new trigger of pemphigus vulgaris? Dermatology. (1997) 195:48–9. doi: 10.1159/000245685 9267738

[101] Golberg O Harman KE. Drug-induced pemphigus. In: Katsambas AD Lotti TM Dessinioti C D’Erme AM, editors. European handbook of dermatological treatments. Berlin, Heidelberg: Springer (2015). p. 725–30. doi: 10.1007/978-3-662-45139-7_72

[102] Tang X Zhang X. Drug-induced pemphigus after six years of treatment with phenytoin and carbamazepine. Int J Dermatol. (2012) 51:485–6. doi: 10.1111/j.1365-4632.2010.04706.x 22320341

[103] Böhm R. OpenVigil Cave-at document . Available online at: https://openvigil.sourceforge.net/doc/openvigil-cave-at-v2.html (Accessed October 30, 2023).

[104] Sakaeda T Tamon A Kadoyama K Okuno Y. Data mining of the public version of the FDA adverse event reporting system. Int J Med Sci. (2013) 10:796–803. doi: 10.7150/ijms.6048 23794943 PMC3689877

