# Supplementary figures and images for: A comprehensive, population level evaluation of previously reported drug triggers of pemphigus highlights immunomodulatory capacity as a common characteristic
Source: Front Immunol. 2025 Jan 21;15:1508129. doi: 10.3389/fimmu.2024.1508129 (PMC11790476; doi:10.3389/fimmu.2024.1508129)

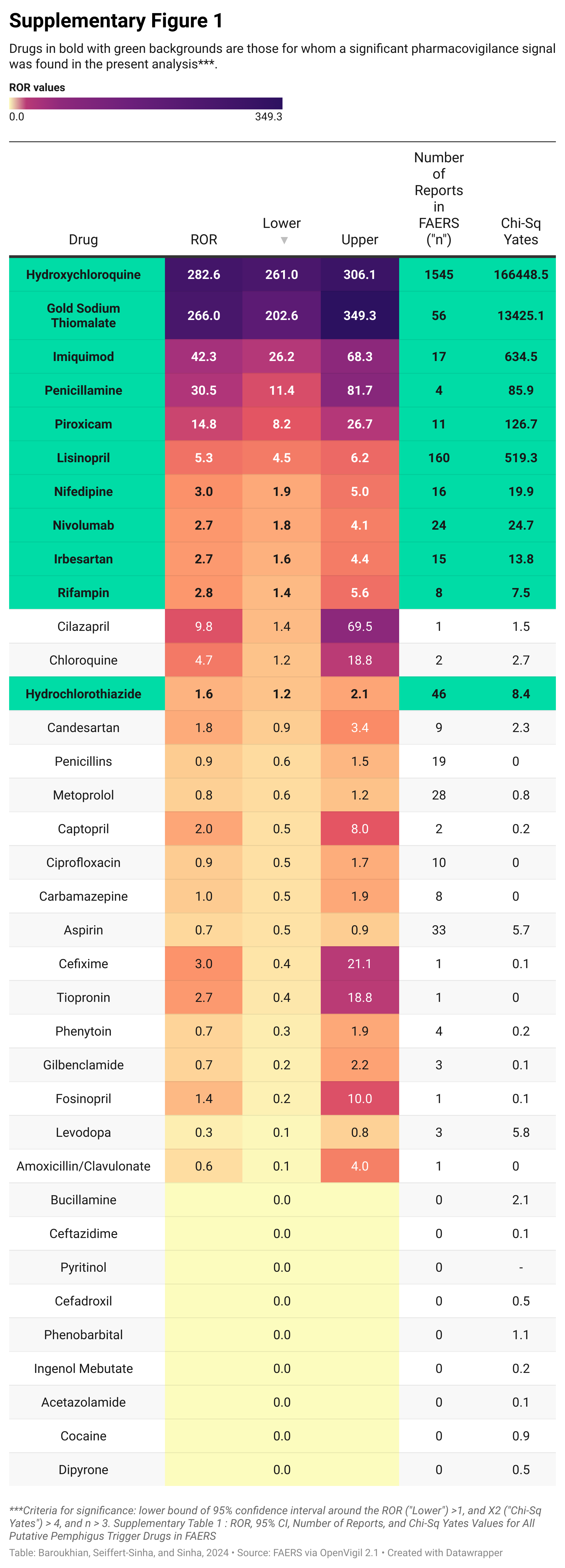

Supplement: Supplementary file 1 [file Image1.png]
